# Assessment of Pharmacokinetic Interaction Between Gefapixant (MK‐7264), a P2X3 Receptor Antagonist, and the OATP1B1 Drug Transporter Substrate Pitavastatin

**DOI:** 10.1002/cpdd.1047

**Published:** 2021-11-24

**Authors:** Jacqueline B. McCrea, Azher Hussain, Bennett Ma, Graigory C. Garrett, Raymond Evers, John E. Laabs, S. Aubrey Stoch, Marian Iwamoto

**Affiliations:** ^1^ Merck & Co. Inc. Kenilworth New Jersey USA; ^2^ Johnson & Johnson Janssen Pharmaceuticals Springhouse Pennsylvania USA; ^3^ Celerion 2420 W. Baseline Road Tempe Arizona USA

**Keywords:** drug‐drug interaction, organic anion transporting polypeptide, chronic cough

## Abstract

Gefapixant (MK‐7264, AF‐219), a first‐in‐class P2X3 antagonist, is being developed as oral treatment for refractory or unexplained chronic cough. Based on in vitro data, gefapixant exerts inhibitory activity on the organic anion transporter (OAT) P1B1 transporter. Therefore, a drug‐drug interaction study evaluating the potential effects of gefapixant on the OATP1B1 drug transporter, using pitavastatin as a sensitive probe substrate, was conducted. An open‐label, 2‐period, fixed‐sequence study in 20 healthy adults 18 to 55 years old was conducted. In period 1, a 1‐mg oral dose of pitavastatin was administered to each participant. After a ≥4‐day washout, in period 2 participants received a 45‐mg oral dose of gefapixant twice daily on days 1 through 4. On day 2 of period 2, pitavastatin was coadministered with the morning dose of gefapixant. Pitavastatin exposures following single‐dose administration with and without multiple doses of gefapixant were similar: geometric mean ratio (90% confidence interval) of pitavastatin area under the plasma concentration–time curve from time 0 to infinity (AUC_0‐∞_) (pitavastatin + gefapixant/pitavastatin alone) was 0.97 (0.93‐1.02). The ratio of pitavastatin lactone AUC_0‐∞_ to pitavastatin AUC_0‐∞_ was also comparable between treatments. Administration of gefapixant and pitavastatin was generally well tolerated, with no safety findings of concern. These results support that gefapixant has a low potential to inhibit the OATP1B1 transporter.

Cough is among the most common symptoms prompting individuals in the United States to visit a physician.^1^, ^2^ For some patients, a cough prompted by conditions such as asthma or gastroesophageal reflux persists despite treatment of the underlying cause^3^ and becomes refractory chronic cough (RCC), defined as lasting >8 weeks.^4^ For other patients, an underlying cause of chronic cough cannot be identified (unexplained chronic cough [UCC]).^5^ The global prevalence of chronic cough is estimated to be approximately 10%.^6^ Therapies for RCC and UCC are currently limited, leaving many patients with significant, untreated morbidity.^7^

Cough is regulated by airway sensory nerves that express the adenosine triphosphate–gated ion channels P2X3.^8^, ^9^ Damaged, stressed, or inflamed tissues release adenosine triphosphate, which is sensed as an urge to cough, resulting in activation of the cough reflex. The P2X3 antagonist, gefapixant (MK‐7264, AF‐219), is an oral compound, currently in clinical development, that has shown promise to suppress cough in patients with RCC or UCC.^10^, ^11^, ^12^

Gefapixant is rapidly absorbed following oral administration, with a time to maximal plasma concentration (t_max_) of 1 to 4 hours; plasma concentration declines rapidly, with an elimination half‐life (t_1/2_) of 6 to 10 hours. Steady state is achieved within 2 days of dosing. The primary route of elimination of gefapixant is renal excretion. Based on in vitro data, gefapixant exerts inhibitory effects on the OATP1B1 transporter. Given that gefapixant may be used chronically and may be coadministered with other medications, including those that are substrates of the OATP1B1 transporter, it is of value to understand the impact of gefapixant on the pharmacokinetics of OATP1B1 substrates.

Pitavastatin, a hydroxymethylglutaryl coenzyme A reductase inhibitor, is a sensitive clinical probe substrate of OATP1B1 activity.^13^ Pitavastatin is transported into the liver primarily by OATP1B1. It is mainly excreted unchanged; about 15% is eliminated in urine and about 79% in feces. Pitavastatin undergoes minimal metabolism via glucuronidation (uridine 5′‐diphospho‐glucuronosyltransferase 1A3 and uridine 5′‐diphospho‐glucuronosyltransferase 2B7) to pitavastatin lactone and is also minimally metabolized by cytochrome P450 (CYP) enzymes (CYP2C9 and to a lesser extent CYP2C8). The mean plasma elimination half‐life is ≈12 hours.^14^ Previously described pharmacokinetic (PK) data for pitavastatin codosed with OATP1B1 inhibitors (such as single‐dose rifampin and cyclosporin) demonstrate that pitavastatin is a sensitive and relatively selective substrate of the OATP1B1 transporter, with increases in pitavastatin area under the plasma concentration–time curve (AUC) and maximum observed plasma concentration (C_max_) of ≈5‐fold or greater observed in the presence of an OATP1B1 inhibitor.^13^, ^15^

To evaluate the potential for gefapixant to cause a clinically relevant drug‐drug interaction (DDI) with medications that are OATP1B1 substrates, a 2‐period, fixed‐sequence study was conducted during which the effects of multiple‐dose gefapixant on the single‐dose pharmacokinetics of pitavastatin in healthy adults was evaluated. In this study, 1 mg of pitavastatin was chosen to provide safety margins for healthy participants. Multiple doses of gefapixant, using the projected clinical dose of 45 mg twice daily, were to approximate steady state before pitavastatin coadministration. Gefapixant administration continued through day 4 to retain inhibitory effects over the PK sampling period. The results of this study are reported here.

## Methods

This study (Protocol MK‐7264‐044) was granted institutional review board approval by Advarra Inc. (Columbia, Maryland). All participants gave written informed consent. The study was conducted in accordance with Good Clinical Practices guidelines and the ethical principles set forth by the Declaration of Helsinki. It was conducted at Celerion Inc. (Tempe, Arizona) between July 25, 2019, and September 5, 2019.

### Participants

Eligible study participants were men or women, 18 to 55 years of age, with a body mass index of 18 to 30 kg/m^2^ who were nonsmokers and in good health based on medical history, physical examination, laboratory safety tests (including negative urine drug and alcohol screen), vital sign measurements, and electrocardiogram (ECG). Eligible female participants were not pregnant or lactating, or were of non–childbearing potential or of childbearing potential using highly effective contraception or abstinence. The study excluded individuals with a history of allergy or of any drug hypersensitivity, especially to gefapixant or pitavastatin, or history, signs, or symptoms of adverse reaction to sulfonamides.

### Study Procedures

The study was a nonrandomized, fixed‐sequence, 2‐period, open‐label, DDI study. In period 1, participants received a single dose of 1 mg of pitavastatin. After a washout period of at least 4 days (based on the ≈12‐hour half‐life of pitavastatin) in period 2, participants received 45 mg of gefapixant twice daily) on days 1 through 4. On day 2 of period 2, a single dose of pitavastatin was coadministered with the morning dose of gefapixant. In both periods, study drugs were administered following an overnight fast of at least 10 hours before morning dosing. Fasting continued for at least 4 hours after dosing on day 1 of period 1 and after the morning dose on day 2 of period 2. Fluids were restricted from 1 hour before until 1 hour after each dose with the exception of 240 mL of water administered with each dose. There were no fasting requirements before the evening dose of gefapixant. Plasma samples were collected for pitavastatin PK analysis before dosing and 0.25, 0.5, 1, 1.5, 2, 3, 4, 6, 8, 12, 16, 24, 32, 48, and 72 hours after dosing with pitavastatin, with or without gefapixant.

Safety was monitored throughout the study by collection of adverse events (AEs) and evaluation of vital signs, ECGs, and clinical laboratory measures (hematology, chemistry, and urinalysis).

### Bioanalytical Analysis

Plasma samples were assayed for pitavastatin and pitavastatin lactone concentrations using a validated liquid chromatography with tandem mass spectrometric method (Pharma Medica Research Inc., Ontario, Canada). The standard calibration range for pitavastatin and pitavastatin lactone was 0.100 to 200 ng/mL. The plasma samples, treated with dipotassium ethylenediaminetetraacetic acid as the anticoagulant, were acidified by addition of a solution of 1.00 M of ammonium acetate pH 5, in a 1:20 ratio. The acidification step is based on analytical methods previously published, which describe the need for stabilization to limit interconversion of pitavastatin lactone to pitavastatin.^16^ Pitavastatin and pitavastatin lactone were extracted from plasma with organic solvents, the organic phase was dried, and the reconstituted samples were transferred for analysis. Processed samples were analyzed by reverse phase chromatography (Prominence UFLC, Shimadzu Corp., Kyoto, Japan; and API 4000, SCIEX, Framingham, Massachusetts) with mobile phases composed of 40% acetonitrile and 0.01% acetic acid. Chromatographic separation was achieved using a C18, 50 × 4.6 mm, 2.6‐μm analytical column. Pitavastatin and pitavastatin lactone were analyzed using positive ion scan mode. The parent‐daughter mass‐to‐charge ion transitions of pitavastatin and its internal standard, pitavastatin‐d_5_, were 422→290 and 427→295, respectively; both had a chromatographic peak retention time of ≈1.4 minutes. The parent‐daughter mass‐to‐charge ion transitions of pitavastatin lactone and its internal standard, pitavastatin lactone‐d_5_, were 404→274 and 409→276, respectively; both had a chromatographic peak retention time of approximately 1.7 minutes. The extraction recoveries of pitavastatin and pitavastatin lactone were 70.3% to 82.0% and 75.7% to 88.7%, respectively. Intraday accuracy of pitavastatin and pitavastatin lactone quality control samples during validation was 97.6% to 104.7% and 96.0% to 109.0% with precision of ≤5.1% and ≤6.2%. The interday study accuracy of the pitavastatin and pitavastatin lactone quality control samples was 97.7% to 99.6% and 97.1% to 99.7% with precision of ≤4.0% and ≤5.2%.

### Pharmacokinetic Analysis

PK parameter values of pitavastatin and pitavastatin lactone were determined from plasma concentration–time data by employing a noncompartmental approach using Phoenix WinNonlin version 7.0 (Certara, Princeton, New Jersey). The parameter values were read into SAS data sets and all descriptive statistics were calculated in SAS version 9.4 (SAS Institute, Cary, North Carolina). Plasma pharmacokinetics were tabulated by analyte and intervention.

PK parameters evaluated included AUC from time 0 to infinity (AUC_0‐∞_), calculated as AUC_0‐last_ + C_est,last_/λ_z_, where AUC_0‐last_ is the plasma AUC from time 0 to the time of last measurable concentration calculated by the “linear‐up/log‐down” variation of the linear trapezoidal method, C_est,last_ is the last estimated detectable concentration, and λ_z_ is the terminal‐phase elimination rate constant estimated from the slope of the concentration vs time profiles (at least 3 consecutive time points in the terminal phase, excluding t_max_, were used for the apparent terminal t_1/2_ determination); C_max_; t_max_; t_1/2_ (calculated as ln(2)/λ_z_); and apparent total plasma clearance of drug (calculated as Dose/AUC_0‐∞_). The AUC_0‐∞_ metabolite to AUC_0‐∞_ parent ratio for pitavastatin lactone to pitavastatin was calculated as (AUC_0‐∞_ pitavastatin lactone/AUC_0‐∞_ pitavastatin) × (MW of pitavastatin/MW of pitavastatin lactone).

### Statistical Methods

For the primary analysis of AUC, assuming a true within‐subject standard deviation of 0.22 for the natural log of AUC_0‐∞_ with n = 20 completed participants, alpha = 0.05, then there is an ≈84% probability that the 90% confidence interval (CI) for the true geometric mean AUC_0‐∞_ ratio (pitavastatin with gefapixant/pitavastatin alone) will fall within 0.80 to 1.25, given that the true geometric mean ratio is 1. In the event that only 18 participants are evaluable for pharmacokinetics, then there is a 79% probability that the 90% CI for the true geometric mean AUC_0‐∞_ ratio (pitavastatin with gefapixant/pitavastatin alone) will be contained in 0.80 to 1.25, given that the true ratio is 1.

Individual pitavastatin PK parameter values were natural log‐transformed and analyzed using a linear mixed‐effects model with treatment as a fixed effect. An unstructured covariance matrix was used to allow for unequal treatment variances and to model the correlation between different treatment measurements within the same participant via the REPEATED statement in SAS PROC MIXED. The Kenward‐Roger method was used to calculate the denominator degrees of freedom for the fixed effects (DDFM = KR).

For plasma AUC_0‐∞_ and C_max_, the least squares mean differences and the 2‐sided 90% CIs for each parameter ([pitavastatin with gefapixant] – [pitavastatin alone]) in the natural log scale were calculated on the basis of the model described above. The least squares mean differences and CIs were then exponentiated to obtain geometric mean ratios and 90% CIs for the true ratios (pitavastatin with gefapixant/pitavastatin alone). Safety data were summarized.

## Results

A total of 20 participants were enrolled in the study. All completed the study per protocol and were included in the analyses. Participant's mean age (years) ± standard deviation was 42.5 ± 7.7 (range, 19‐54). Mean (range) weight and body mass index were 70.5 kg (51.5‐95.7) and 26.3 kg/m^2^ (19.6‐29.9), respectively. A total of 70% (n = 14) were women, 75% (n = 15) were of Hispanic ethnicity, and all were of White race.

### Effect of Gefapixant on Pitavastatin PK

**Figure 1:**
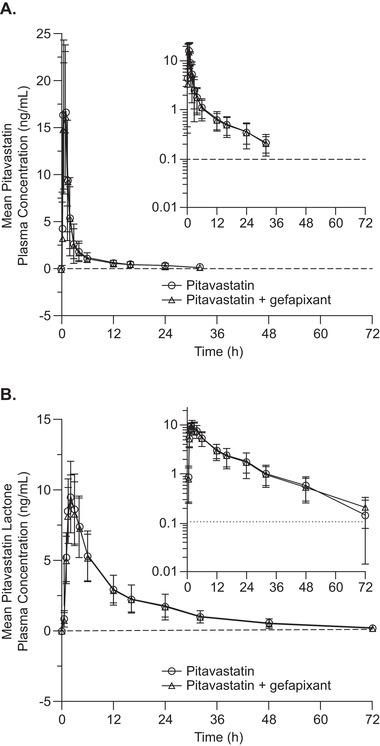
Arithmetic mean (± standard error) plasma concentration–time profiles of pitavastatin (A) and pitavastatin lactone (B) following administration of a single oral 1‐mg pitavastatin dose to healthy participants, without (○) or with (△) multiple oral doses of 45‐mg gefapixant twice daily for 4 days (N = 20). The insets show the data plotted on a semilogarithmic scale.

Gefapixant had no meaningful effect on plasma pitavastatin or pitavastatin lactone concentration versus time profiles (Figures 1A and 1B). Pitavastatin concentrations at 48 and 72 hours after dosing pitavastatin alone or with gefapixant are not presented in Figure 1A because the majority of the values were less than the lower limit of quantitation. Pitavastatin and pitavastatin lactone exposures (AUC_0‐∞_ and C_max_) following single‐dose administration of pitavastatin with and without multiple doses of gefapixant were similar (Table 1). Pitavastatin lactone exposure and the ratio of pitavastatin lactone AUC_0‐∞_ to pitavastatin AUC_0‐∞_ were also similar during both treatments (Table 1). The median t_max_, geometric mean t_1/2_ and apparent total plasma clearance of pitavastatin were comparable between the 2 treatments, as were the median t_max_ and geometric mean t_1/2_ of pitavastatin lactone (Table 1).

**Table 1:** Pharmacokinetic Parameter Values of Pitavastatin and Pitavastatin Lactone in Healthy Participants Following Oral Administration of a Single Dose of 1‐mg Pitavastatin Without or With Multiple Doses of 45‐mg Gefapixant Twice Daily (N = 20)

	Pitavastatin (N = 20)				Pitavastatin + Gefapixant (N = 20)					
	Pitavastatin		Pitavastatin Lactone		Pitavastatin		Pitavastatin Lactone		Pitavastatin	Pitavastatin Lactone
Parameter	GM (95% CI)	AM (SD)	GM (95% CI)	AM (SD)	GM (95% CI)	AM (SD)	GM (95% CI)	AM (SD)	GMR^a^ (90% CI)	
AUC_0‐∞_, ng • h/mL	44.3 (36.1‐54.3)	48.5 (22.2)	116 (98.2‐136)	123 (45.5)	43.1 (35.4‐52.5)	47.0 (21.1)	117 (99.9‐137)	124 (43.6)	0.97 (0.93‐1.02)	1.01 (0.97‐1.05)
C_max_, ng/mL	17.8 (14.7‐21.6)	19.3 (7.6)	9.42 (8.34‐10.7)	9.73 (2.53)	17.5 (14.8‐20.7)	18.6 (6.9)	8.92 (8.01‐8.94)	9.15 (2.12)	0.98 (0.90‐1.07)	0.95 (0.91‐0.98)
t_max_,^b^ h	0.78 (0.50‐1.03)	…	2.01 (1.50‐4.03)	…	0.50 (0.50‐1.0)	…	2.00 (1.50‐3.00)	…	…	…
t_1/2_,^c^ h	14.6 (28.0)	15.1 (4.6)	15.0 (20.4)	15.3 (3.30)	12.6 (28.5)	13.1 (4.2)	16.8 (23.0)	17.2 (3.65)	…	…
CL/F,^c^ L/h	22.6 (45.7)	24.6 (10.2)	…		23.2 (43.9)	25.1 (9.7)	…	…	…	…
RAUC_0‐∞_	…	…	2.73 (2.38‐3.12)	2.83 (0.78)	…	…	2.83 (2.49‐3.23)	2.94 (0.82)	…	1.04 (1.01‐1.08)

AM, arithmetic mean; AUC_0‐∞_, area under the plasma concentration–time curve from time 0 to infinity; C_max_, maximum observed plasma concentration; CI, confidence interval; CL/F, apparent total plasma clearance; GM, geometric mean; RAUC_0‐∞_, ratio AUC_0‐∞_ metabolite to AUC_0‐∞_ parent; SD, standard deviation; t_1/2_ = apparent terminal half‐life; t_max_, time when C_max_ was first observed.

AUC % extrapolated (median; minimum‐maximum): pitavastatin (6.7; 4.1‐14.5), pitavastatin with gefapixant (6.0; 3.8‐12), pitavastatin lactone (3.8; 1.4‐11.6), and pitavastatin lactone with gefapixant (4.5; 1.4‐7.3).

^a^ GM ratio of values from pitavastatin + gefapixant/pitavastatin.

^b^ Median (minimum‐maximum) reported for t_max_.

^c^ Values shown under “GM” are GM and geometric percent coefficient of variation.

### Safety

Gefapixant in combination with pitavastatin was generally well tolerated. The overall incidence of AEs across treatments was 75% (n = 15), with 5 (25%) participants reporting ≥1 AEs following pitavastatin alone (period 1), 12 (60%) following gefapixant alone (day 1 period 2), and 7 (35%) following pitavastatin + gefapixant (days 2 to 4 period 2). No serious AEs or deaths occurred during this study. The most frequently reported AEs in the study each occurred in 3 (15%) participants following any given intervention; these were headache following pitavastatin + gefapixant, and nausea, fatigue, dysgeusia, or somnolence, following gefapixant alone. All AEs reported in the study were considered by the investigator to be mild to moderate in severity and resolved by study completion. No clinically meaningful relationships were observed between changes in clinical laboratory values, vital signs, or safety ECG parameters and interventions.

## Discussion

Gefapixant is a P2X3 receptor antagonist in development as a treatment for unexplained or refractory chronic cough. Based on in vitro data, gefapixant exerts inhibitory activity on the OATP1B1 transporter with a half maximal inhibitory concentration of 35 μM obtained following a 30‐minute preincubation. Given that the R value was nearing the cutoff value listed in the in vitro Food and Drug Administration DDI guidance,^17^ a clinical DDI study was conducted with an expectation that the potential of gefapixant perpetrating an OATP1B1‐mediated DDI would be minimal. In the DDI study reported here, the 90% CI of the geometric mean ratio of the AUC_0‐∞ _of pitavastatin dosed with gefapixant to the AUC_0‐∞_ of pitavastatin dosed alone ranged from 0.93 to 1.02, demonstrating that the pharmacokinetics of pitavastatin are not meaningfully altered by gefapixant. Gefapixant also had no meaningful effect on pitavastatin C_max_ (see Table 1). In addition, there was no meaningful effect of gefapixant on the pharmacokinetics of the major pitavastatin metabolite, pitavastatin lactone. The DDI data presented within are consistent with the low risk that was predicted based on the in vitro data.

Gefapixant was generally well tolerated during this study. The reported AEs following gefapixant with or without pitavastatin were generally consistent with reports from other studies.^10^, ^11^, ^12^

With regard to potential study limitations, only a single dose of 1 mg pitavastatin was used. The pitavastatin PK profile is adequately characterized following single‐dose administration, and impact of OATP1B1 inhibition is readily seen, as demonstrated in prior single‐dose pitavastatin OATP1B1 inhibition clinical trials.^13^, ^15^ A fixed‐sequence design was implemented to limit operational complexity due to the multiple doses of gefapixant required in the combination period 2. However, because of this study design, period and treatment effects are confounded.

## Conclusion

There is low potential of gefapixant to affect the disposition of coadministered drugs that are substrates of the OATP1B1 transporter.

## Conflicts of Interest

J.B.M., A.H., B.M., G.C.G., R.E., S.A.S., and M.I. are current or former employees of Merck Sharp & Dohme Corp., a subsidiary of Merck & Co., Inc., Kenilworth, New Jersey, and may own stock/stock options in Merck & Co., Inc.. J.E.L. declares no conflicts of interest.

## Funding

Funding for this study was provided by Merck Sharp & Dohme Corp., a subsidiary of Merck & Co., Inc., Kenilworth, New Jersey.

## Author Contributions

All authors provided final approval of the version to be published and agree to be accountable for all aspects of the work in ensuring that questions related to the accuracy or integrity of any part of the work are appropriately investigated and resolved.

## Data Availability

The data‐sharing policy, including restrictions, of Merck Sharp & Dohme Corp., a subsidiary of Merck & Co., Inc., Kenilworth, New Jersey, is available at http://engagezone.msd.com/ds_documentation.php. Requests for access to the clinical study data can be submitted through the Engage Zone site or via email to dataaccess@merck.com.
